# Anthropogenic Disturbance Can Determine the Magnitude of Opportunistic Species Responses on Marine Urban Infrastructures

**DOI:** 10.1371/journal.pone.0022985

**Published:** 2011-08-03

**Authors:** Laura Airoldi, Fabio Bulleri

**Affiliations:** 1 Dipartimento di Biologia Evoluzionistica Sperimentale and Centro Interdipartimentale di Ricerca per le Scienze Ambientali in Ravenna, Università di Bologna, Ravenna, Italy; 2 Department of Earth and Environmental Sciences, Wesleyan University, Middletown, Connecticut, United States of America; 3 Dipartimento di Biologia, Università di Pisa, CoNISMa, Pisa, Italy; 4 Dipartimento di Science Botaniche, Ecologiche e Geologiche, Università di Sassari, Sassari, Italy; Dalhousie University, Canada

## Abstract

**Background:**

Coastal landscapes are being transformed as a consequence of the increasing demand for infrastructures to sustain residential, commercial and tourist activities. Thus, intertidal and shallow marine habitats are largely being replaced by a variety of artificial substrata (e.g. breakwaters, seawalls, jetties). Understanding the ecological functioning of these artificial habitats is key to planning their design and management, in order to minimise their impacts and to improve their potential to contribute to marine biodiversity and ecosystem functioning. Nonetheless, little effort has been made to assess the role of human disturbances in shaping the structure of assemblages on marine artificial infrastructures. We tested the hypothesis that some negative impacts associated with the expansion of opportunistic and invasive species on urban infrastructures can be related to the severe human disturbances that are typical of these environments, such as those from maintenance and renovation works.

**Methodology/Principal Findings:**

Maintenance caused a marked decrease in the cover of dominant space occupiers, such as mussels and oysters, and a significant enhancement of opportunistic and invasive forms, such as biofilm and macroalgae. These effects were particularly pronounced on sheltered substrata compared to exposed substrata. Experimental application of the disturbance in winter reduced the magnitude of the impacts compared to application in spring or summer. We use these results to identify possible management strategies to inform the improvement of the ecological value of artificial marine infrastructures.

**Conclusions/Significance:**

We demonstrate that some of the impacts of globally expanding marine urban infrastructures, such as those related to the spread of opportunistic, and invasive species could be mitigated through ecologically-driven planning and management of long-term maintenance of these structures. Impact mitigation is a possible outcome of policies that consider the ecological features of built infrastructures and the fundamental value of controlling biodiversity in marine urban systems.

## Introduction

Marine landscapes have been altered globally by the introduction of a variety of man-made infrastructures, such as seawalls, dykes, breakwaters, groynes, jetties, pilings, bridges, artificial reefs, offshore platforms, and marine energy installations [1]–[4]. In Europe, 22000 km^2^ of the coastal zone is covered in concrete or asphalt, and about 50% of the Mediterranean shorelines bordering Spain, France, and Italy are dominated by artificial infrastructures (more than 1500 km), most of which are developed for harbours and ports ([5] and references therein). In the USA, armouring covers more than 50% of the coastline in some estuaries and bays ([4] and references therein); overall, about 21% of the 759 km coastline of Florida and 12% of the 1763 km coastline of California have been altered by armouring, or addition of bulkheads, revetments, or other coastal infrastructures. Similarly, in the Western Pacific, 27% of the coastline in Japan [6] and more than 50% of the shores of Sydney Harbour [7] have been altered by either coastal infrastructure or armouring. It is expected that marine infrastructures will further proliferate in response to burgeoning coastal populations, expansion of coastal cities, and greater threats from climate change, storm surges and sea level rise [3], [4], [8].

As marine artificial substrata support many species colonising epibiota, it has been suggested that these artificial substrata may represent adequate mimics of natural hard-bottom habitats [8]–[9], or valuable surrogates for the habitats that they replace [10]–[11]. However, studies from different regions of the world and from different types of artificial infrastructures suggest that artificial surfaces do not function as natural rocky habitats [12]–[15], and often introduce surfaces and species that are extraneous to the natural environments [16]–[18]. These studies document differences in the structure of assemblages inhabiting artificial infrastructures compared to nearby natural rocky shores [15], [19]–[22], including low species and genetic diversity [23]–[25], rarity of particular functional groups, such as large grazers and predators [24], different ecological processes [15], [26]–[30], and assemblages representative of the early stages of succession, comprising opportunistic, weedy, and invasive species [15], [17], [31]–[34].

In terrestrial systems, the prevalence of opportunistic and invasive forms in urban areas has often been attributed to severe disturbances, typical of human-dominated systems [35]–[39], such as those linked with soil use, deforestation, fires, construction activities, gardening, and recreation, among others. Disturbances (both natural and anthropogenic) are also implicated in the prevalence of opportunistic and non-indigenous species in a variety of marine systems [e.g. 40–44]. However, the role of anthropogenic disturbances in facilitating the spread of opportunistic, and invasive species on artificial infrastructures is relatively unexplored [32]. Marine artificial infrastructures tend to be subjected to high levels of disturbances from both natural factors [e.g. storms and sediment scour, which are especially intense on infrastructures constructed for protection against erosion and flooding [21], [45] and anthropogenic factors [e.g. harvesting and trampling, which are intense on many artificial infrastructures due to their accessibility from highly tourist beaches [46]–[47].

Another common and particularly severe form of disturbance, which is unique to artificial infrastructures, is represented by maintenance works. The failure rates of coastal infrastructures as a result of scour, undermining, outflanking, overtopping, and battering by storm waves are relatively high, and there is an ongoing need for repair and maintenance during the lifetime of the structure [48]. The ecological consequences of repair and maintenance for the biota of artificial infrastructures are currently unknown.

We analysed how the expansion of opportunistic and invasive forms on coastal defence infrastructures along the Italian side of the north Adriatic Sea (Italy) could be influenced by the continued repair and maintenance, We measured (1) the extent, frequency, and timing of occurrence of periodical maintenance, and (2) quantified the response trajectories of assemblages following maintenance. Since breakwaters introduce both sheltered (i.e. on the landward sides) and exposed (on the seaward sides) substrata that support different assemblages [17], [31]–[32], we also tested (3) whether maintenance has different effects in these habitats. Finally, since recovery of assemblages is influenced by the mix of propagules, spores, and larvae present in the water column at the time at which free space becomes available [49]–[50], we experimentally tested(4) if disturbance imposed at different times of the year could influence the recovery of assemblages, and control the abundance of opportunistic and invasive forms. Results are used to identify possible management strategies in an effort to improve the ecological outcomes of artificial marine infrastructures.

## Materials and Methods

### Study area

The study was conducted along ∼50 km of coast of the north-east Adriatic Sea (Italy), from Punta Marina (44°45′N, 12°29′E) to Cesenatico (44°20′ N, 12°40′ E). The area is characterised by flat sandy substrate, with moderate exposure to wave action and an average tidal amplitude of ∼80 cm. Average surface sea temperature varies between 8°C in the winter and 24°C in the summer.

**Figure 1:**
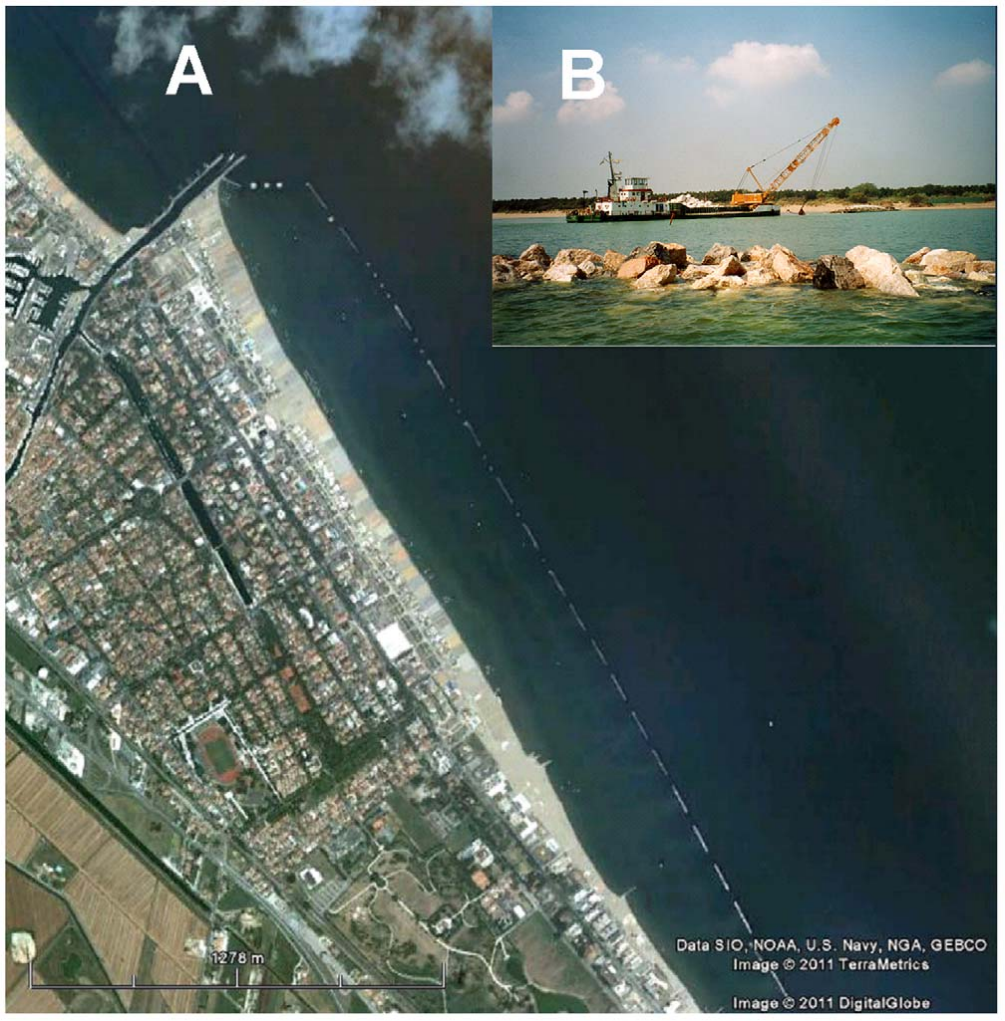
Human-made, coastal defence infrastructures at Cesenatico, along the Italian shores of the north Adriatic sea. A) View of the study area and breakwaters from Google Earth. B) View of a breakwater under maintenance (photo L. Airoldi).

This region is severely urbanised [5], [51]. Over the past 60 years, a wide variety of marine artificial infrastructures have been built along >60% of this sedimentary coastline (Fig. 1A), including >100 km of breakwaters and groynes, >60 km of seawalls and >40 km of jetties. Breakwaters (Fig. 1A), built with large blocks of quarried rock (1–3 m across), are deployed at ∼100–250 m from the shore, have an average length of 100–150 m, and extend ∼2–3 m above and below the Mean Low Water Level (MLWL). These breakwaters thus provide both subtidal and intertidal surfaces for colonisation by benthic organisms [17], [32], along both wave-sheltered (landward) and wave-exposed (seaward) habitats.

The study focused on assemblages at low-shore levels (−10 to +20 cm relative to MLWL). At these levels on the shore, ephemeral and weedy macroalgae (*Ulva* spp.), filamentous forms, the introduced green alga *Codium fragile* ssp. *tomentosoides*), and biofilms (comprising coatings of microalgae, juvenile stages of macroalgae and silt) are the dominant floral taxa on many breakwaters. Other dominant faunal taxa include mussels (*Mytilus galloprovincialis*), oysters (*Ostrea edulis* and the introduced *Crassostrea gigas*), barnacles (*Chthamalus* spp. and *Balanus perforatus*), and the limpet *Patella caerulea.* Detailed descriptions of these assemblages can be found in [17], [32], [46], [52]–[53].

### Disturbance regime

Many coastal infrastructures are constructed using quarried rocks that move during sea storms [54]. Mobile rocks are subjected to stresses that can cause breakage, size reduction, or dislodgement. Maintenance, therefore, often involves the addition of new quarried rocks over large portions of the defence structures to repair damages from storms (Fig. 1B). We monitored maintenance interventions to breakwaters over 3 years, from January 2001 to December 2003, at 25 reference breakwaters. The breakwaters were selected at random along 50 km of coast. We recorded: (1) the occurrence of maintenance interventions; (2) the time of the year at which interventions were carried out, and (3) measured the extension of the damage caused by interventions, as the proportion (% of the total length) of the breakwater affected by the addition of new rocks.

### Effects of maintenance

In April 2002, extensive maintenance was carried out at Cesenatico (Fig. 1A). About one third of the breakwaters at this locality were maintained through the addition of new blocks over >70% of the surface. We randomly selected 4 breakwaters among those that had undergone maintenance; 4 additional breakwaters, that were not maintained, were randomly selected as controls. Sampling started in May 2002, after a storm stabilised the new blocks and made sampling safe from risks associated with the potential overturning of blocks. Eight replicate quadrats (20 × 20 cm), randomly placed at least 1 m apart, were sampled visually at low-shore levels on both the landward and seaward sides of each breakwater, by using a frame with 25 sub-quadrats. A score from 0 (i.e. absence) to 4% (i.e. occupation of the entire surface) was given to each taxon in each sub-quadrat, and the total cover was obtained by summing over the entire set of sub-quadrats [55]. Organisms were generally identified to species level, or grouped into higher taxonomic or morphological groups when unequivocal identification in the field was not possible. Sampling was repeated in August 2002, and January and May 2003 to identify the recovery time trajectory. Although the study was planned to run until complete recovery, 2 control breakwaters were maintained in May 2003 and as a result, the experiment was terminated.

Effects of maintenance in relation to the exposure and location of the assemblages were analysed using PERMANOVA [56] on Bray-Curtis similarity coefficients, calculated using fourth-root transformed data to preserve information on relative covers of species, while reducing differences in scales among variables. For the analysis, 9999 unrestricted random permutations of residuals were used to generate *P*-values. The analysis included the factors: date (random, 4 levels), treatment (fixed, 2 levels: maintained *vs* non-maintained control), exposure (fixed, 2 levels: landward *vs* seaward), and breakwater (random, 4 levels, nested within treatment). A metric multi-dimensional scaling (MDS) plot, calculated on a matrix of centroids in Bray-Curtis space (PCO; [56]) for each combination of date, treatment, breakwater, and exposure, was used to visualise patterns in multivariate data. Effects on the most abundant taxa were also analysed individually using Analysis of Variance (ANOVA), including the same factors as in the multivariate analysis. Cochran's *C* test was used to assess the assumption of homogeneity of variances and data were transformed when necessary. Student – Newman – Keuls (SNK) tests were used for a posteriori comparisons of means (57).

### Effects of the timing of disturbance

A manipulative experiment was carried out at Cesenatico to test whether the timing at which maintenance, or other extensive disturbances are imposed affects the recovery of the assemblages and the dominance by weedy macroalgae, and whether any such effects varied between the landward and seaward sides of breakwaters and among breakwaters. In March 2003, 16 blocks >3 m apart, were randomly selected on both the landward and seaward sides of 3 randomly chosen breakwaters (100s m apart), that had not been maintained during the past 3 years. Subsets of 4 blocks were randomly assigned to each of 3 different disturbance times (April 2003, August 2003, January 2004), and controls (undisturbed blocks). Assemblages were removed from the entire surface of each block by means of paint-scrapers and brushes, in order to simulate the effects of large disturbances such as those from maintenance. Blocks assigned to different times of disturbance were marked for later relocation.

Assemblages on treatment and control blocks were sampled in May 2004, as most of the macroalgae on these breakwaters are annual species, characterised by a peak in abundance during the spring-summer. Four quadrats (20 × 20 cm) were randomly placed on each block and sampled visually, using the same technique described in the previous section. No further sampling was possible after this date, as breakwaters were subjected to unscheduled maintenance works.

The effects of disturbances applied at different times, and in relation to the exposure and location of the assemblages, were analysed using PERMANOVA. The analysis included the factors treatment (4 levels, fixed), breakwater (3 levels, random), and exposure (fixed, seaward *vs* landward), crossed to each other, and the factor block (4 levels, random), nested in the interaction treatment × exposure × breakwater. A MDS plot, calculated on a matrix of centroids in Bray-Curtis space for each combination of treatment, breakwater, and exposure, was used to visualise patterns in multivariate data. The same design was used to evaluate the response of weedy macroalgae to manipulative conditions using ANOVA.

## Results

From 2001 to 2003, approximately 52% of the monitored breakwaters were maintained only once, 16% were maintained twice, and 32% were not maintained. Almost 80% of the repairs were made during spring months, from mid-March to mid-June. Maintenance opened a substantial amount of bare space, through both the addition of new surfaces and the disruption of extant assemblages by overturning blocks (Fig. 1 B). The extent of bare space just after maintenance varied from ∼30–40% on the seaward sides of the breakwaters to >70% on the landward sides.

### Effects of maintenance

**Figure 2:**
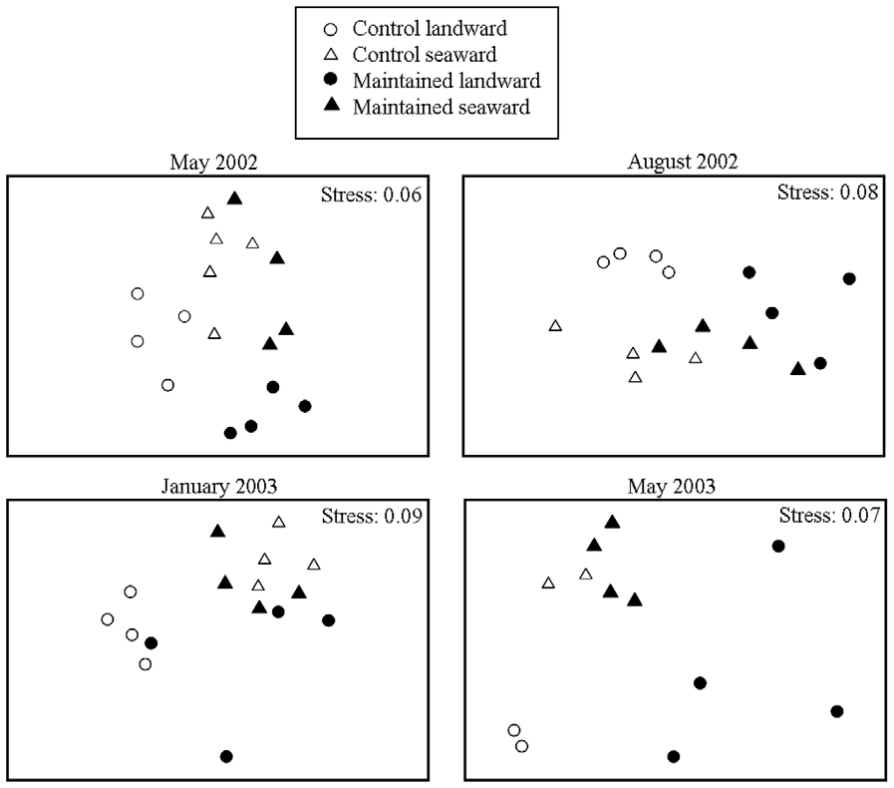
Two-dimensional plots of principal coordinate axes (unconstrained metric multi-dimensional scaling, MDS). The MDS shows ordination of centroids of assemblages at the landward and seaward sides of replicated control (non-maintained) and maintained (March 2002) breakwaters at Cesenatico in May 2002, August 2002, January 2003 and May 2003. There were 4 control and 4 maintained breakwaters, except for May 2003, when there were only 2 control breakwaters. Analyses were based on Bray-Curtis dissimilarities after 4th-root transformation of cover data. Stress values lower than 0.10 indicate that the ordination is good and that the interpretation of patterns in 2 dimensions is reliable.

There were significant effects of disturbance due to maintenance works that varied among dates of sampling (Table 1). Assemblages on maintained breakwaters were different from those on non-maintained breakwaters after 1 (May 2002), and 4 months (August 2002) after the time of disturbance (Fig. 2, Table 1). Despite the lack of significant effects of maintenance works in January 2003, low-shore assemblages on maintained and non-maintained breakwaters differed in May 2003, more than a year after maintenance had occurred (Fig. 2, and Table 1). Disturbance had consistently greater effects on assemblages on the landward than on the seaward sides, as shown by the significant interaction treatment × exposure (Table 1).

**Table 1:** Effects of maintenance in relation to the exposure and location of the assemblages on the breakwaters.

Source	df	MS	Pseudo-F	Pair-wise tests
Date = D	3	24310	13.49***	
Treatment = T	1	68385	5.52**	
Exposure = E	1	69816	6.49**	
Breakwater (T) = B (T)	6	5415	3.01***	**D x T**
D x T	3	7912	4.39***	May02: M ≠ C, Aug02: M ≠ C,
D x E	3	7223	3.02**	Jan03: M = C, May03: M ≠ C
T x E	1	21632	3.98**	**T x E**
D x B (T)^a^	16	1802	1.93***	Landward: M ≠ C, Seaward: M = C
E x B (T)	6	4408	1.84*	
D x T x E	3	1925	0.80	
D x E x B (T)^a^	16	2392	2.56**	
Residual	420	934		

The analysis is a PERMANOVA (17 variables, 4th-root transformed data) comparing assemblages between maintained ( = M) and non-maintained, control ( = C) breakwaters (Treatment, fixed factor), among dates of sampling (May and August 2002, January and May 2003, random factor; in May 2003, two control breakwaters were missing), between landward and seaward sides (Exposure, fixed factor) and among Breakwaters (4 levels, random factor nested in Treatment). ^a^ Term has one or more empty cells. *  =  *P*<0.05; **  =  *P*<0.01, *** = *P*<0.001.

**Figure 3:**
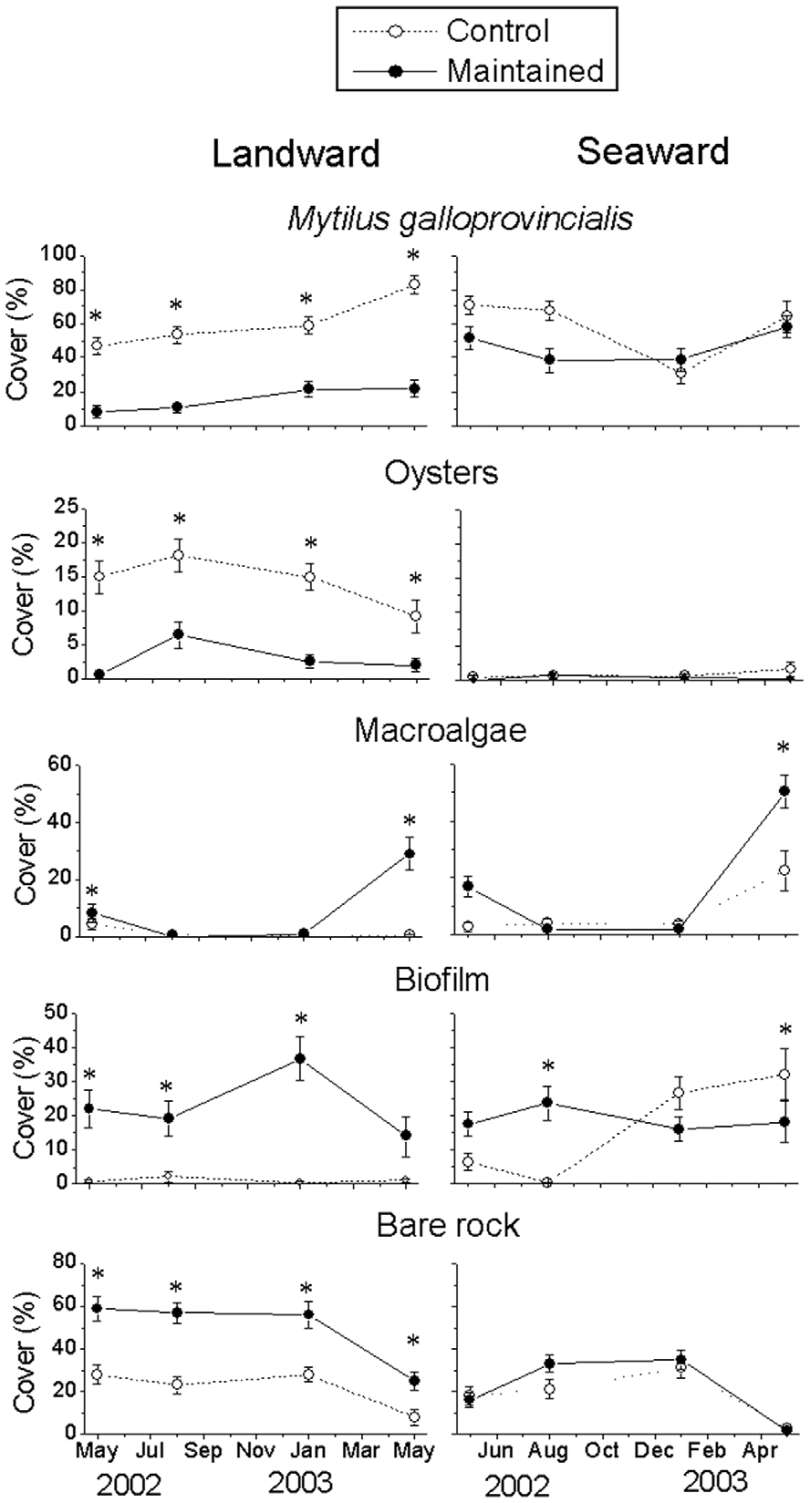
Abundance of most abundant taxa on the study breakwaters at Cesenatico. Data are average percent covers ±1 SE (*n* = 32, 8 replicate plots for each of 4 breakwaters; in May 2003 *n* = 16 for controls, 8 replicate plots for each of 2 breakwaters) of *Mytilus galloprovincialis*, oysters (a mixture of *Ostrea edulis* and *Crassostrea gigas* difficult to separate by visual sampling), macroalgae (mainly *Ulva* spp., *Codium fragile* ssp. *tomentosoides* and filamentous forms), biofilm (a coating of microalgae, juvenile stages of macroalgae and silt) and bare rock (rock non occupied by visible macroscopic forms) at the landward and seaward sides of control (non-maintained) and maintained (April 2002) breakwaters, in May 2002, August 2002, January 2003 and May 2003. Asterisks indicate significant differences between assemblages on maintained and control breakwaters as indicated by *a posteriori* SNK tests.

On the landward sides, maintenance caused the immediate loss of mussels and oysters and an increase in the availability of bare space, much of which was immediately occupied by a biofilm coating and, to a lesser extent, by macroalgae (Fig. 3; see Supporting Information S1 for details of the univariate analyses). In May 2002, 1 month after the maintenance, bare rock and biofilm together comprised >80% of primary substrata, compared to <30% on control breakwaters. Bare rock and biofilm cover decreased in May 2003, when macroalgae became the dominant taxa on maintained breakwaters (Fig. 3). Macroalgae, which comprised ephemerals, such as *Ulva* spp., filamentous forms, and the invasive species *Codium fragile* ssp. *tomentosoides*, were virtually absent from the landward sides of non-maintained breakwaters.

On the seaward sides, maintenance had less severe effects on mussels, initially increasing the cover of the biofilm by only 10–20% (Fig. 3). However, similar to results observed on the landward sides, macroalgae also increased significantly on the seaward sides following the maintenance (Fig. 3), reaching up to 50% cover on maintained breakwaters, compared to <20% on non-maintained breakwaters by May 2003. The increase in macroalgae on both the landward and seaward sides of breakwaters was particularly interesting because it occurred not only during the spring-summer, immediately following the repairs, but also during the following spring, one year later.

### Effects of the timing of disturbance

In May 2004, the landward sides of breakwaters were extensively covered by a thin layer of sediment, possibly as a consequence of nearby beach nourishment. Increased sediment deposition probably negatively affected filter-feeding organisms (i.e. mussels and oysters), reducing their% cover compared to the beginning of the experiment. Nonetheless, it was still possible to clearly detect differences between blocks that had been disturbed at different times.

**Figure 4:**
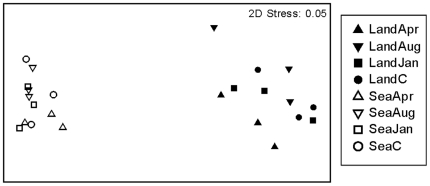
Two-dimensional plots of principal coordinate axes (metric multi-dimensional scaling, MDS). The MDS shows ordination of centroids of assemblages at the landward and seaward sides of each of 3 breakwaters for each time of disturbance (D1 =  April 2003, D2 =  August 2003, D3 =  January 2004, C =  unmanipulated plots). Analyses were based on Bray-Curtis dissimilarities after 4th-root transformation of data collected in May 2004. A Stress value of 0.05 indicates that the ordination is excellent and that the interpretation of patterns in 2 dimensions is highly reliable.

In May 2004, assemblages on blocks disturbed in April and August 2003 were still significantly different from those on control blocks (Table 2, Fig. 4). In contrast, there were no detectable differences between assemblages disturbed in January 2004 and the controls (Table 2, Fig. 4), despite the shorter period of time which had elapsed. These patterns did not vary among breakwaters or between the landward and seaward sides, despite the large natural variation at this scale, and between these habitats (Table 2).

**Table 2:** Effects of disturbance applied at different times and in relation to the exposure and location of the assemblages on the breakwaters.

Source of variation	df	MS	Pseudo-F
Treatment = T	3	5404.1	9.11**
Exposure = E	1	2.95	11.14^*^
Breakwater = B	2	33544	16.50**
T x E	2	1745.5	0.94
T x B	6	593.1	0.29
E x B	2	26496	13.03
T x E x B	6	1844.3	0.91
Block (T x E x B)	72	2033.6	4.27^**^
Residual	288	475.75	
pair-wise tests: Treatment			
Comparison	*t*	*P*	
D1, D2	18.355	0.001	
D1, D3	1.6832	0.118	
D1, C	2.6654	0.032	
D2, D3	4.7585	0.005	
D2, C	2.9879	0.021	
D3, C	1.6012	0.141	

The analysis is a PERMANOVA (14 variables, 4th-root transformed data) comparing assemblages among breakwaters, between landward and seaward sides, among treatments (removal in April 2003 = D1, August 2003 = D2 and January 2004 = D3; unmanipulated plots  = C) and among blocks, 10 months after the initiation of the experiment. * =  *P*<0.05; ** =  *P*<0.01.

**Figure 5:**
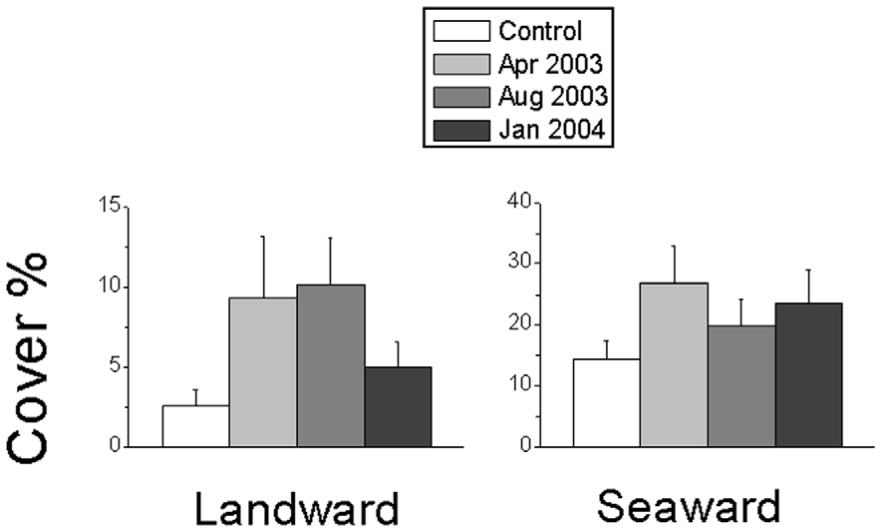
Abundance of macroalgae in the experimental treatments at Cesenatico. Data are average percent covers ±1 SE (*n* = 36, 4 replicate plots for each of 4 blocks for each of 3 breakwaters) of macroalgae (mainly *Ulva* spp. and *Codium fragile* ssp. *tomentosoides*) at the landward and seaward sides of breakwaters for each time of disturbance (April 2003, August 2003, January 2004, Control  =  unmanipulated plots).

The abundance of weedy macroalgae varied according to the time at which the disturbance was applied (F_3,72_ = 4.58, *P* = 0.054, see Supporting Information S2 for details of the univariate analyses). The cover of macroalgae (mostly *Ulva* spp.) on blocks disturbed in April 2003 was approximately double that on controls, on both the landward and seaward sides (Fig. 5). Blocks disturbed in August 2003 also supported a greater cover of macroalgae (mostly the invasive *Codium fragile* ssp. *tomentosoides*) than controls, but this was only significant on the landward sides (Fig. 5). Conversely, blocks disturbed in January 2004 developed macroalgal coverage comparable to undisturbed controls. The cover of other species greatly varied among replicates and blocks, and it was not possible to identify a clear response to the treatment (Supporting Information S2).

## Discussion

Our results clearly indicate that intense human disturbance can be a major determinant of the spread of opportunistic species on artificial coastal infrastructures. Maintenance - which is an extreme disturbance - caused a significant decrease in the cover of dominant species such as mussels and oysters and enhanced the cover of opportunistic organisms such as biofilms, and weedy and invasive macroalgae. These effects were particularly prevalent on the landward (sheltered) sides of breakwaters, while assemblages on the seaward (exposed) sides were only moderately affected by the maintenance.

The different responses to disturbance observed between the landward and seaward sides of the breakwaters can be interpreted as a result of a combination of factors. Firstly, disturbance *per se* was more severe and persistent on the landward sides. The new blocks added to the breakwaters (e.g. Fig. 1B) were initially unstable and were overturned by wave action, rolling most frequently towards the landward sides. Secondly, the structure of dominant mussel beds also differed between the landward and seaward sides of breakwaters [32], [46]: on the landward sides, mussels were generally larger in size and formed a multi-layered matrix, whilst on the seaward sides, smaller individuals formed mono-layered beds [58], which tend to be less susceptible to dislodgement by mechanical disturbances [59]–[60]. Finally, on the seaward sides, mussels, which were the dominant space occupiers, recovered more quickly. Efficient colonisation of space in exposed habitats may result from greater rates of recruitment of larvae or faster growth of individuals, due to greater provision of food particles in enhanced water flow [61]–[62].

The manipulative experiment confirmed that the high cover of weedy macroalgae, often observed on artificial infrastructures in the study region, can result from severe disturbances, such as those from maintenance. In addition, it showed that the cover of these species varied according to the timing at which the disturbance was applied. The cover of macroalgae increased by ∼100% on all substrata disturbed in April, and on landward substrata disturbed in August, while no increase was observed when the disturbance was applied in January. The macroalgal assemblage was mostly composed of opportunistic forms, representing an early stage of succession, and invasive species. In the study area, *Ulva* spp. are amongst the earliest colonisers on new infrastructures, sometimes attaining up to 100% cover 2 months post-construction [17].

Although the experiment was not specifically designed to test if recovery trajectories differed depending on the timing of disturbance, our results suggest that the time at which the disturbance is applied could also influence the duration of recovery to reference conditions. Assemblages disturbed in January 2004 were similar to controls after only 4 months, while assemblages disturbed earlier (in April and August 2003) still differed from controls, despite the longer period of time which had elapsed. This suggests a non - linear relationship between recovery and time elapsed since disturbance. A possible reason why substrata disturbed in January recovered more quickly than substrata disturbed at other times could be related to the different recruitment peaks of mussels and macroalgae in this region. Mussels tend to recruit in late winter to early spring [63], while macroalgae recruit in the spring to late summer [17]. Therefore, the effects of disturbances occurring in winter, when macroalgae are less likely to monopolise space and mussel recruitment is about to start, could be short-lasting in this system. In contrast, disturbances occurring in spring-summer could result in a longer-term persistence of macroalgae. The mortality of mussels in spring 2004 (prior to our sampling in May), probably caused by the large deposition of sediments, may have increased the similarity between assemblages on blocks disturbed shortly before (January) and those on control blocks. However, a similar response on the sheltered sides of breakwaters, little affected by the enhanced deposition of sediments, indicates that this was not the primary mechanism responsible for the patterns observed.

Our results are consistent with recent work showing how the spatio-temporal variation in the effects of disturbance is a key factor in determining the success of opportunistic and invasive species in marine systems [41], [44], [64]. This has important implications for the design and management of hard coastal infrastructures and, more broadly, for the conservation of coastal areas, under increasing anthropogenic pressures. Ecological considerations in the design of marine infrastructures tend to focus on construction materials, surface texture, and habitat complexity, as engineering options to enhance the ecological value of these artificial substrata [65]–[68]. Here we show that, in the long run, the project lifetime and required maintenance are clearly one of the most crucial factors affecting the composition, abundance, and distribution of species that colonise the infrastructures. For any new infrastructure introduced into the marine environment, it will take time for mature assemblages to develop [69]–[71]. Furthermore, any following maintenance, or other analogous severe disturbances, will lead to the assembly of communities that represent early successional stages. Importantly, the magnitude of this impact and the capability of the system to recover will largely depend on the scale (spatial and temporal) of the disturbance. This has some significant policy implications, in terms of incorporating aspects of the ecology of the system into decisions regarding the timing of major maintenance and repairs.

Marine urban infrastructures are often located in harsh environments and their lifetime would be significantly reduced without routine maintenance or periodic repairs. At the same time, our results suggest that approaches to maintenance could be improved, for example, by carrying out repair interventions at specific times or in a way that reduces their impacts. For example, in the study area, the impact of maintenance on assemblage dynamics would be reduced if interventions are carried out over the winter rather than spring. Higher operational costs would be largely offset by both environmental and economical benefits. Optimising maintenance would reduce the development of weedy macroalgal species. Macroalgal blooms and their associated detritus washed up on beaches are a common problem in urban coastal areas, causing nuisance to recreational and tourist activities and leading to expensive removal operations [72]–[74]. Additionally, sound management of maintenance activities would reduce the likelihood of the establishment and/or spread of non-indigenous marine species, with indirect benefits for fisheries and aquaculture. Marine man-made infrastructures are particularly sensitive to invasions by non-indigenous species [31]-[34]. Our results, in accordance with those of previous studies [32], [46], suggest that the great invasibility of artificial infrastructures may be due to the severe disturbances to which they are generally exposed. Indeed, prolonged availability of unoccupied space, or other resources generated by human disturbances are considered one of the main factors facilitating the establishment of non-indigenous species [75].

### Conclusions

Mitigation of impacts of marine infrastructures is a possible outcome of policies that explicitly consider the ecological features of artificial habitats and recognise the fundamental value of managing biodiversity in urban settings. We advocate that there is a need for ecological based planning and management of urban artificial infrastructures. Understanding the functioning of these novel habitats will be key to improving their design and management and, hence, to mitigate their negative impacts and enhance their contribution to marine biodiversity and ecosystem functioning.

## Supporting Information

Supporting Information S1 ANOVAs on the effects of date, maintenance works, exposure and breakwater on the percentage cover of unoccupied space, biofilm, macroalgae and invertebrates.
(DOC)

Supporting Information S2 ANOVAs on the effects of Breakwater, Exposure and Treatment on the percentage cover of unoccupied space and dominant taxa.
(DOC)
