# Supplementary material for: Anthropogenic Disturbance Can Determine the Magnitude of Opportunistic Species Responses on Marine Urban Infrastructures
Source: PLoS One. 2011 Aug 3;6(8):e22985. doi: 10.1371/journal.pone.0022985 (PMC3149642; doi:10.1371/journal.pone.0022985)
Supplement: Supporting Information S1 — ANOVAs on the effects of date, maintenance works, exposure and breakwater on the percentage cover of unoccupied space, biofilm, macroalgae and invertebrates. (DOC) [file pone.0022985.s001.doc]

*Supporting Information S1. ANOVAS on the effects of Date of sampling (May and August 2002, January and May 2003, random factor; in May 2003, two control breakwaters were missing), Treatment (maintained vs non-maintained breakwaters, fixed factor), Exposure (seaward vs landward, fixed factor) and Breakwater (4 levels, random factor nested in Treatment) on the percentage cover of unoccupied space, biofilm, macroalgae, Mytilus galloprovincialis and oysters. * P < 0.05; ** P < 0.01 ***; P < 0.001; NS = not significant. Pooling procedures were used according to Underwood (1997) [57], and terms not significant at P < 0.25 were eliminated from the analysis when appropriate.*

| Source of variation |  | Bare rock |  | Biofilm | |  | Macroalgae | |  | *M. galloprovincialis* | |  | Oysters |  |
| --- | --- | --- | --- | --- | --- | --- | --- | --- | --- | --- | --- | --- | --- | --- |
|  | df | MS | *F* | MS | *F* | | MS | *F* | | MS | *F* | | MS | *F* |
| Date = D | 3 | 20666.00 | 21.39*** | 20.40 | 4.26* | | 97.57 | 60.65*** | | 7300.00 | 4.85* | | 3.05 | 1.83 |
| Treatment = T | 1 | 29890.13 | no test | 173.13 | no test | | 22.21 | no test | | 102413.50 | no test | | 113.69 | no test |
| Exposure = E | 1 | 31203.14 | no test | 178.62 | no test | | 74.82 | 4.36 | | 25308.98 | 2.88 | | 176.87 | no test |
| Breakwater (T) = B(T) | 6 | 2104.774 | 2.18 | 17.14 | 3.58* | | 6.04 | 3.75* | | 2646.38 | 1.76 | | 2.12 | 1.28 |
| D x T | 3 | 1133.08 | 1.17 | 48.53 | 10.13*** | | 19.38 | 12.04*** | | 2691.25 | 1.79 | | 0.82 | 0.49 |
| D x E | 3 | 1809.05 | 1.24 | 39.65 | 4.12* | | 17.17 | 4.62* | | 8783.97 | 4.51* | | 2.69 | 2.64 |
| T x E | 1 | 18939.45^a^ | 6.79* | 84.27 | no test | | 0.72 ^b^ | 0.35 | | 36572.45^b^ | 11.11* | | 76.87 ^a^ | 34.65** |
| D x B(T) | 16 | 966.10 | 1.97* | 4.79 | 1.05 | | 1.61 | 1.69 | | 1504.36 | 1.88* | | 1.66 | 2.56*** |
| E x B(T) | 6 | 2790.63 | 1.91 | 16.51 | 1.71 | | 1.78 | eliminated | | 2516.75 | eliminated | | 2.22 | 2.18 |
| D x T x E | 3 | 292.33 | eliminated | 30.85 | 3.20* | | 7.62 | 2.05 | | 3292.80 | 1.69 | | 1.04 | eliminated |
| D x E x B(T) | 16 | 1459.52 | 2.98*** | 9.63 | 2.11** | | 3.72 | 3.91*** | | 1946.44 | 2.43*** | | 1.02 | 1.57 |
| Residual | 216 | 489.53 | 4.57 | 0.95 | 799.52 | | 0.65 |  | |  |  | |  |  |
|  |  |  |  |  |  | |  |  | |  |  | |  |  |
| Transformation |  | None |  | Square root | |  | Ln(x+1) |  | | None |  | | Ln(x+1) |  |
| Cochran’s test |  | NS |  | NS |  | | NS |  | | NS |  | | NS |  |

^a^ Tested against the terrn E x B(T); ^b^ Tested against the terrn D x T x E
