# Supplementary material for: Anthropogenic Disturbance Can Determine the Magnitude of Opportunistic Species Responses on Marine Urban Infrastructures
Source: PLoS One. 2011 Aug 3;6(8):e22985. doi: 10.1371/journal.pone.0022985 (PMC3149642; doi:10.1371/journal.pone.0022985)
Supplement: Supporting Information S2 — ANOVAs on the effects of Breakwater, Exposure and Treatment on the percentage cover of unoccupied space and dominant taxa. (DOC) [file pone.0022985.s002.doc]

*Supporting Information S2. Analyses on the effects of Breakwater, Exposure and Treatment (removal of assemblages in April, August 2003, January 2004 and controls) on the percentage cover of dominant taxa and unoccupied space. * P < 0.05; ** P < 0.01 ***; P < 0.001; NS = not significant.*

| Source of variation |  | *M. galloprovincialis* | |  | Macroalgae |  | Biofilm |  | Unoccupied space | |  |
| --- | --- | --- | --- | --- | --- | --- | --- | --- | --- | --- | --- |
|  | df | MS | *F* | | MS | *F* | MS | *F* | MS | *F* | |
| Breakwater = B | 2 | 6690.39 | 5.79** | | 14.04 | 6.59 | 22801.25 | 32.54*** | 20415.58 | 19.95*** | |
| Exposure = E | 1 | 135657.05 | 27.80* | | 215.52 | 191.63** | 22801.25 | 1.00 | 310907.30 | 18.82* | |
| Treatment = T | 3 | 549.78 | 0.71 | | 4.13 | 4.58^a^ | 81.44 | 1.00 | 1078.08 | 1.34 | |
| B x E | 2 | 4880.16 | 4.22* | | 1.12 | 0.53 | 22801.25 | 32.54*** | 16516.72 | 16.14*** | |
| B x T | 6 | 777.46 | 0.67 | | 0.90 | 0.42 | 81.44 | 0.12 | 802.29 | 0.78 | |
| E x T | 3 | 1524.13 | 0.90 | | 2.14 | 0.89 | 81.44 | 1.00 | 830.40 | 0.53 | |
| B x E x T | 6 | 1696.25 | 1.47 | | 2.40 | 1.13 | 81.44 | 0.12 | 1561.71 | 1.53 | |
| Block (B x E x T) | 72 | 1155.81 | 4.85*** | | 2.13 | 3.47*** | 700.66 | 5.53*** | 1023.25 | 3.99*** | |
| Residual | 288 | 238.09 | 0.61 | |  | 126.64 | 256.36 |  |  |  | |
|  |  |  |  | |  |  |  |  |  |  | |
| Transformation |  | None |  | | Ln(x+1) |  | None |  | None |  | |
| Cochran’s test |  | * |  | | NS |  | *** |  | NS |  | |

^a^ 0.05 < *P* < 0.06
